# Surgical management of benign prostate hyperplasia in Nigeria: open prostatectomy versus transurethral resection of the prostate

**DOI:** 10.11604/pamj.2021.39.165.24767

**Published:** 2021-07-02

**Authors:** Chimaobi Gideon Ofoha, John Edoka Raphael, Nuhu Kutan Dakum, Samaila Ibrahim Shu'aibu, Julius Akhaine, Isaac Musa Yaki

**Affiliations:** 1 Department of Surgery, Jos University Teaching Hospital (JUTH), Jos, Nigeria,; 2 College of Health Sciences, University of Jos, Jos, Nigeria,; 3 Department of Surgery, College of Health Sciences, University of PortHarcourt, PortHarcourt, Nigeria,; 4 Department of Urology, University of PortHarcourt Teaching Hospital, PortHarcourt, Nigeria

**Keywords:** Benign prostate hyperplasia, open prostatectomy, TURP, complications, surgical management

## Abstract

**Introduction:**

transvesical (open) prostatectomy (OP), an invasive surgical procedure, is a common form of treatment offered to patients with benign prostatic enlargement in emerging economies. Recently, there has been an increase in the use of electrosurgical means in treating benign prostate hyperplasia in our environment, especially transurethral resection of the prostate (TURP). This study compares the perioperative, short-term outcomes and complications of open prostatectomy and TURP.

**Methods:**

the records of men who had prostatectomies (OP and TURP) from Jan 2016 to Dec 2019 for prostate gland less than 80g were reviewed. The patients´ age, size of the prostate gland, duration of surgery (mins), blood transfusion, clot retention, length of catheterisation (days), hospital stay (days), postoperative infection, postoperative incontinence, reoperation, bladder neck stenosis and urethral stricture were analysed.

**Results:**

fifty-nine patients were studied. Twenty-nine patients had OP, while 30 had TURP. Mean age for OP was 63.8 (SD 7.2) years, while for TURP is 68.5 (SD 8.0) years (p=0.019). The mean for length of catheterisation for OP vs TURP was 9.1 (SD 3.7) vs 3.3 (SD 1.03) days (p=0.001), mean hospital stay was 9.6 (SD 4.1) and 4.7 (SD 2.2) days (p=0.001) for OP and TURP while duration of surgery (mins) for OP and TURP was 106.7 (SD 15.2) vs 53.8 (SD 14.0) minutes (p=0.001). The blood transfusion rate was 13.8% and postoperative incontinence 13.8% in OP, while in the TURP group, the reoperation rate was 3.3% and urethral stricture at a rate of 3.3%. Overall complications showed no statistical difference (p=0.462) between the two groups.

**Conclusion:**

the patients who underwent TURP had shorter hospital stay, shorter duration of surgery and catheterisation, and less frequently required blood transfusion compared to those who had open prostatectomy. However, reoperation rate was higher compared to open prostatectomy. The overall complication was fewer with TURP, but this is not statistically significant; hence both forms of surgical therapy remain relevant in a poor resource setting.

## Introduction

Benign prostatic hyperplasia is a non-malignant enlargement of the prostate gland. It is a common disease that affects ageing men and may cause lower urinary tract symptoms [1,2]. These symptoms impair quality of life and can result in renal dysfunction [3]. After the age of forty, the prevalence of benign prostatic hyperplasia (BPH) increases in men. Histopathological observations in autopsy studies have shown a prevalence of 8%, 50% and 80% in the 4^th^, 6^th^ and ninth decades of life [4,5]. Recurrent acute urinary retention, recurrent urinary tract infection, failed voiding trials, recurrent gross haematuria and renal insufficiency secondary to obstruction are indications for prostatectomy. Other indications for prostatectomy include failed medical therapy, a desire to terminate medical therapy and financial constraints associated with medical therapy [6-8].

Surgical management of benign prostate hyperplasia includes open prostatectomy (transvesical prostatectomy), transurethral holmium laser ablation of the prostate, transurethral holmium laser enucleation of the prostate, holmium laser resection of the prostate, photoselective vaporisation, transurethral incision of the prostate, transurethral vaporisation of the prostate and transurethral resection of the prostate (TURP) [9-11]. Open prostatectomy (OP), an invasive surgical procedure, is a common form of treatment offered to patients with prostatic enlargement in emerging economies [8,12]. Open surgery is determined by patients´ presentation, anatomy and the experience of the surgeon.

However, recently, there has been an increase in the use of electrosurgical means in treating benign prostatic hyperplasia in our environment, especially transurethral resection of the prostate (TURP), a minimally invasive endourological procedure that employs a monopolar or bipolar current-based resection of the prostate [13-15]. Despite the growing trend in the use of electrosurgical means in the treatment of benign prostate enlargement, the dearth of equipment, power supply and training has been a challenge in our environment. Consequently, open prostatectomy remains the primary modality of treatment for comparable prostate volumes [8,16,17].

This study compares the perioperative, short-term outcomes and complications of open prostatectomy and transurethral resection of the prostate.

## Methods

**Study design:** this is a hospital-based retrospective study comparing open prostatectomy and transurethral resection of the prostate.

**Study population:** men who had prostatectomy (open prostatectomy and transurethral resection of the prostate) for prostate gland less than 80g from January 2016 to December 2019 were studied.

**Data collection:** the records of the patients who were followed up for one year after prostatectomy were retrieved and recorded in a proforma. Patients with incomplete records or lost to follow-up were excluded. Patient evaluation included history, physical examination, including digital rectal examination and investigation (serum level of creatinine, full blood count, prostate-specific antigen (PSA) assay, urine analysis and urine culture; abdominal ultrasonography to assess the kidneys, ureters, urinary bladder and determine the prostate size and other changes in the urinary tract). The patients´ age, size of the prostate gland (grams), duration of surgery (mins), blood transfusion, clot retention, length of catheterisation (silicon catheter) in days, hospital stay (days), postoperative infection, postoperative incontinence, reoperation, bladder neck stenosis and urethral stricture were analysed.

**Statistical analysis:** all data obtained were subjected to statistical analysis using the Statistical Package for Social Sciences (SPSS) version 23 statistical software. Results were represented in tables. Chi-square, Fisher´s exact test and T-test were used for analysis with assistance of a statistician. P-value of <0.05 was considered significant.

**Ethical considerations:** informed consent was obtained from all the patients. The institutional review board (IRB) permits retrospective studies.

## Results

Fifty-nine patients who had prostatectomy were studied. Twenty-nine patients had open prostatectomy, while thirty had transurethral resections of the prostate. Mean age for open prostatectomy was 63.8 (SD 7.23) years, while for TURP, it was 68.5 (SD 8.0) years (p=0.019) (Table 1). Shows the age grouping for the patients. The mean prostate size (grams) for OP was 65.9 (SD 20.0) while for TURP was 59.7 (SD 19.7) (P= 0.239).

**Table 1:** the age grouping for the patients

Age group	Open prostatectomy	Transurethral resection of the prostate	Total	p
50-59	8 (27.6%)	3 (10.0%)	11 (18.6%)	0.175
60-69	13 (44.8%)	14 (46.7%)	27 (45.8%)	
≥70	8 (27.6%)	13 (43.3%)	21 (35.6%)	
Total	29 (100%)	30 (100%)	59 (100%)	

The different mean for the length of catheterisation (postoperative), duration of hospital stay and duration of surgery is shown in Table 2. The TURP group had a shorter hospital stay, length of catheterisation and shorter duration of surgery. In patients who had open prostatectomy, blood transfusion was required in four patients (13.8%) with postoperative incontinence occurring in 4 (13.8%), while there were no transfusions and no postoperative incontinence in the TURP group. In the TURP group, the reoperation rate was 3.3%, bladder neck stenosis 3.3% and urethral stricture 3.3%, whereas none were recorded in the OP group (Table 3). Clot retention and postoperative infection occurred in both groups.

**Table 2:** comparison of length of catheterisation, hospital stay and surgery duration for open prostatectomy against transurethral resection of the prostate

Variables	Open prostatectomy (mean ± SD)	Transurethral resection of the prostate (mean ± SD)	P-value
Length of catheterization (days)	9.1 (3.7)	3.3 (1.0)	0.001
Hospital stay (days)	9.6 (4.1)	4.7 (2.2)	0.001
Duration of surgery (Mins)	106.67 (15.3)	53.8 (14.0)	0.001

**Table 3:** comparison of complications for open prostatectomy vs transurethral resection of the prostate

Complications	Open prostatectomy (n=29)	Transurethral resection of the prostate (n=30)	Total (n=59)	P-value
Blood transfusion	4 (13.8%)	0 (0.0)	4 (6.8%)	0.052
Clot retention	2 (6.9%)	2 (6.7%)	4 (6.8%)	1.000
Epididymo-orchitis	3 (10.3%)	5 (16.7%)	8 (13.6%)	0.706
Postoperative incontinence	4 (13.8%)	0 (0.0)	4 (6.8%)	0.052
Reoperation	0 (0.0)	1 (3.3%)	1 (1.7%)	1.000
Bladder neck stenosis	0 (0.0)	1 (3.3%)	1 (1.7%)	1.000
Urethral stricture	0 (0.0)	1 (3.3%)	1 (1.7%)	1.000

The overall complication rate for open prostatectomy was 9.0%, while for TURP, it was 6.7% (p=0.462).

## Discussion

The surgical management of benign prostate hyperplasia is dynamic and new methods of treatment are emerging rapidly [18,19]. Most of these treatment modalities are technology-driven and are not readily accessible to patients in resource-poor countries. The gold standard for surgical treatment of BPH is TURP and is increasingly available in our environment though open prostatectomy remains widely practiced [15-17]. In this study, the overall complication rate for TURP was 6.7%, while for OP, it was 9.0% (p-value=0.462). Though the TURP group had fewer overall complications compared to the OP group, this was not statistically significant (p-value=0.462).

Blood transfusion requirement during surgery serves as a surrogate for intraoperative blood loss. Open prostatectomy is perceived to be associated with significant blood losses and allogeneic blood transfusions [20]. This is in keeping with the finding in this study, where 13.8% of the patients who had open prostatectomy required blood transfusion while none of the patients in the TURP group had a transfusion. Salako *et al*. had a similar transfusion rate of 13.8%, while Oranusi *et al*. recorded 18% transfusion rate for patients who had open prostatectomy. While none of the patients in the preliminary experience with monopolar transurethral resection of the prostate in Nigeria had blood transfusion, Alhasan *et al*. and Liu *et al*. had transfusion rates of 0.8% and 10.8%, respectively [13,15,21]. Though the index study did not record blood transfusion in the TURP arm, other studies have shown, albeit low transfusion rates, this should be taken into consideration while performing TURP.

The reoperation rate was 3.3% in the TURP group, while none was recorded in the open prostatectomy group. In a comparative study of open prostatectomy and TURP, the reoperation rate for TURP was 16.3%, while none of the patients in the open prostatectomy group had reoperation [22]. A large-scale, contemporary, nationwide analysis confirmed the higher reoperation rate after TURP compared to open prostatectomy [23]. This is not surprising, as the higher reoperation rate in TURP could be attributed to an average resected prostate tissue weight of 25.8g, which is 54% of gland volume [24].

Urethral stricture occurred in 3.3% in the TURP group, while none occurred in the OP group. The rate of urethral stricture varies from 2.2% to 9.8% in the literature for TURP [25], which is similar to the finding in this study. Other workers have noted the occurrence of urethral stricture in both groups of patients though injuries to the urethra were considerably less frequent with open prostatectomy [26-28]. Instrumentation, urethral injury and inadequate lubrication may account for the higher incidence of urethral stricture in TURP. The low rate of urethral stricture in OP could be explained by the use of silicone catheters, which obviated the effect of toxic catheters on the urothelium.

Postoperative incontinence occurred only in the OP group in this study (13.8%). Other workers have noted postoperative incontinence while performing OP [29,30]. Damage to the external sphincter is the most frequent cause of postoperative incontinence following open prostatectomy. Apical dissection is a blind procedure and inadvertent injury can occur if the apex of the prostate is not gently teased off. Low incontinence rate in TURP can be attributed to the fact that the exact location of the external sphincter can be checked repeatedly during apical resection. Avoiding resection of the veru, which is the landmark for the external sphincter ensures continence postoperatively [31].

Invariably, endoscopic procedures are associated with a short hospital stay. The median hospital stay in this study was shorter for patients who had TURP than for patients who had OP with p=0.001. OP is an invasive procedure hence the longer hospital stay in this group of patients. Other studies have shown similar findings [15-17]. It is a known possibility that extended hospital stay is associated with complications: nosocomial infection, deep vein thrombosis, adverse drug reactions and need for social care, as well as the economic implication of prolonged hospital stay [32].

The mean length of catheterization and duration of surgery for OP compared to TURP was longer in this study (p=0.001, respectively). Similarly, studies on OP and TURP have shown that TURP is consistently associated with shorter length of catheterisation and operative time [15,22,33]. Longer operative time is associated with multiple postoperative complications. This impacts negatively on the patient and increases morbidity [34,35].

Benign prostate hyperplasia is a disease of ageing men, typically begins at the age of forty and the prevalence increases as men age [4]. In this study, the mean age for patients who had OP was 63.8 (SD 7.2) years, while for TURP 68.5 (SD 8.0) years (p=0.019). There was a bias for TURP for older patients, probably because they had more comorbidities hence the preference for a less invasive procedure. The limitations of the study include the small sample size and retrospective nature. A prospective study with a large sample size will give a better insight into the surgical outcome in these patients.

## Conclusion

The patients who underwent TURP had shorter hospital stay, shorter duration of surgery and catheterisation and less frequently required blood transfusion compared to those who had open prostatectomy. However, reoperation rate was higher compared to open prostatectomy. The overall complication was fewer with TURP, but this was not statistically significant; hence both forms of surgical therapy remain relevant in a poor resource setting.

### What is known about this topic

- Benign prostatic hyperplasia is a non-malignant enlargement of the prostate gland;
- Complications because of obstruction due to benign prostate hyperplasia are indications for surgical intervention.

### What this study adds

- The advantages of TURP include short hospital stay, short duration of surgery, less transfusion requirement and short duration of catheterisation;
- The overall complication though fewer with TURP, was statistically insignificant.
